# Thank you to our 2025 reviewers

**DOI:** 10.1016/j.eclinm.2026.103780

**Published:** 2026-01-23

Ali Abbara

Aula Abbara

Ebrahim Abbasi

Lilian Abbo

Rehab Mahmoud Abd El-Baky

Hilal Abdessamad

Midhat H Abdulreda

Ryuichiro Abe

Askar Abiltayev

Eynav Elgavish Accortt

Arianna Aceti

Antoine Adenis

Julien Adjedj

Annelies Aerssens

Jai Prakash Agarwal

Emelia Agblevor

Kingsley Agho

Selidji Todagbe Agnandji

Antonella Agodi

Suleiman Idris Ahmad

Tomohiko Ai

Mark Ainsworth

Olalekan Aiyegbusi

Sheikh Mohammad Fazle Akbar

Shahin Akhondzadeh

Ibrahim Akin

Ziyad Al-Aly

Uazman Alam

Ahu Alanya

Peter Albers

Kassandra Alcaraz

Thierry Alcindor

Tomas Aleman

Kefyalew Alene

Ala Ali

Shabbir Alibhai

Alaa Aljabali

Eman Alkhalawi

Iman Almansour-Alzamil

Hesham Al-Mekhlafi

Fady Alnajjar

Annie Aloysius

Othman Al-Sawaf

Daniel Altmann

Rodabe Amaria

Alexandre Campos Moraes Amato

Namasivayam Ambalavanan

Jayakrishna Ambati

Alain Amstutz

Anandakumar Amutha

Anna Anderson

Peter Anderson

Emanuele Angelucci

Emmanuel Antonarakis

Richard Aplenc

Jason Appleton

Aaron Aragaki

Christine Årdal

Jann Arends

Andrew Argent

Mustafa Arici

Paula Aristizabal

Doruk Arslan

Andrea Gottsegen Asnes

Kaitlyn Atkins

Thomas Atkinson

Shebli Atrash

Mihir R Atreya

Nadine Attal

Koichi Azuma

Peter Baade

Lone Baandrup

Tomohisa Baba

Robert Bachmann

Brian Badgwell

Sunil Badve

Ahmed BaHammam

Anthony D Bai

Shasha Bai

Xiaoyin Bai

Stephen Bain

Ahad Bakhtiari

Abigail Baldridge

Amaris Karin Balitsky

Tala Ballouz

Amitava Banerjee

Aduragbemi Banke-Thomas

Rani Bansal

Liang Bao

Jair Bar

Marlies Bär

Aleksandra Barac

Louise Barbier

Stefano Barco

Ricardo Barini

Hanmant V Barkate

Frédéric Baron

Aluisio Barros

Alfred Bartolucci

Joshua I Barzilay

Saurav Basu

Ibrahim Batal

Yuri Battaglia

Francisco Jose Bautista

Mohsen Bayati

David Beard

Luke Beardon

Richard Beasley

Pilar Beato

Thomas Beblo

Jürg Beer

Ibtissem Nour Elhouda Bekhti

Can Ebru Bekircan-Kurt

Asim Belgaumi

Anna Bellizzi

M Bencic

Yossef S Ben-Porath

Wendie A Berg

Zwi Nisan Berneman

Luca Bertolaccini

Natascia Bertoncelli

Marc Besselink

Katharina Beyer

Christopher Beynon

Vivek Bhadri

Praveen Bharti

Amarnath Bhide

Nazim Bhimani

Francois Clement Bidard

Julia Bielicki

Laura Luisa Bielinski

Candice Biernesser

Geert Jan Biessels

Lore Billiauws

Jack Birch

Einar Bjornsson

Ian Blanchard

Rolf Blauenfeldt

Katherine Bline

Daniel Blumberger

Cauane Blumenberg

Sandeep Bodduluri

Magdalena Bofill

Noémie Boillat-Blanco

Daniel Bolliger

Ralph Bolman III

Giulia Bonavina

Jesper Bonde

Hector Bonilla

Staja Booker

Christopher Booth

Claudio Borghi

Andrea Borondy Kitts

Josep Maria Borras

Elizabeth Boskey

Farid Boubred

Matthieu Boucher

Eric Bouffet

Franck Bourdeaut

Anders Boyd

Maria Bragesjö

Carolyn Bramante

Randall Brand

Birgitte Brandstrup

Lars Christian Haugli Braten

Kees Braun

George Bray

Alana Brennan

Emilio Bria

Matthias Briel

Michael Briga

Tess Bright

Carlo Briguori

Luc Pierre Brion

Inken Brockow

Amy M Brower

Jacques Brown

Thomas Gordon Brown

Monika Bruggemann

Joren Buekers

Diana Buist

Avram Bukhbinder

Danilo Buonsenso

Johan Burisch

Gerrit Burkhardt

Zahid Butt

Robert A Byrne

Ronan Cahill

Mingyan Cai

Xiaoling Cai

Lionel Cailhol

Raffaella Calati

Clifton Callaway

Peter Campochiaro

Daniel Candotti

Michel Canis

Suzanne Cannegieter

Mario Enrico Canonico

Anna Cantarutti

Bin Cao

Yubin Cao

Yunshan Cao

Giovanni Caocci

Gabriel Capella

Filipe Sousa Cardoso

Waldemar Carlo

Arturo Carpio

Alice S Carter

Ben Carter

Jorge Castillo

Jessica Catchpole

Wim Ceelen

Luigi Celio

Paige E Cervantes

Krishnaraj Chadaga

Napasri N Chaisinanunkul

Roger Chammas

Bernard Chan

Joe Kwun Nam Chan

Raymond Chan

Sherry Kit Wa Chan

Shiao-Yng Chan

Wing Lok Chan

Chih-hsiang Chang

Ling-sai Chang

Suhua Chang

Wing Chung Chang

Dipayan Chaudhuri

Nanying Che

Cheng Chen

David Chen

Dawei Chen

Hongda Chen

Hui-Sheng Chen

Jeng-Wen Chen

Jie Chen

Minglong Chen

Minjiang Chen

Mu-Hong Chen

Simiao Chen

Tao Chen

Tao Chen

Wei Chen

Wen Chen

Xiaojun Chen

Yajun Chen

Yu-Chen Chen

Xingxing Shelley Cheng

Ying Cheng

Ankie Tan Cheung

Ching-Lung Cheung

Ka Shing Cheung

Li Cheung

Yee Tak Derek Cheung

Daryl Kai Ann Chia

David Chipanta

Chao Hua Chiu

Peter Ka-Fung Chiu

Kang-Ho Choi

Christos Chouaid

Rashikh Choudhury

Konstantinos Chouliaras

Elaine Chow

Robert Christensen

Min-Hsiang Chuang

Wan–Long Chuang

Carlos Cifuentes-González

Sara Cingarlini

Umit Cirakli

José Miguel Cisneros

Yacouba Cissoko

Jomme Claes

Gavin R C Clark

Richard Clarke

Paola Clauser

John Cleland

Tammy Clifford

Pierluigi Cocco

Hiram Cody

Ricardo Cohen

Roger Cohen

Romain Cohen

Valeria C Cohran

Tim Colbourn

Robert Colebunders

Michael Colla

Simon Collins

Elizabeth Halsted Connors

Pierfranco Conte

Matthew Cooperberg

Andrew Copas

Vladmir Claudio Cordeiro de Lima

David R Cornblath

Rosie Cornish

Wesley M Correll-King

Samuele Cortese

Chandler Cortina

Theodore Cory

Dominique Costagliola

Jacquelyn Crane

Elizabeth A Cromwell

Robert Cross

Iain Crossingham

Mark Crowther

Guillermo Cuervo

Yu Cui

Daniel Culver

Ivan Čundrle

David A Cunningham

Endre Czeiter

Min Dai

Mingshen Dai

Shona Dalal

Alexander Danser

Antoine Dara

Fabrizio D'Ascenzo

Stephen F Daugherty

Jade Davies

Neil Davies

Robert Davis

Andrew Day

Parijat De

Rudolf de Boer

Martin H De Borst

Jan Tjeerd H N de Faber

Francesca De Felice

Nick de Jonge

Elisa de Lazzari

Kelly M de Ligt

Dirk De Ruysscher

Juan B De Sanctis

Paolo de Simone

Armando De Virgilio

Annelou L C De Vries

Nicolas Deconinck

Marloes Dekker Nitert

Anthony Delaney

Geoff Paul Delaney

Rodrigo Delfino

Julio Delgado

Maria Demarco

Guofang Deng

Ting Deng

Nicolau DePaula

Lauranne Derikx

Meenakshi Devidas

Chandra Sekhar Devulapalli

Klodian Dhana

Shyamali Dharmage

Keertan Dheda

Vincenzo Di Lazzaro

Francesco Di Lorenzo

Massimo Di Maio

Mario Di Palma

Betty Diamond

Julia B Dickson-Gómez

María Díez-Campelo

Joakim Dillner

Ellis C Dillon

John Dillon

Andrea Di-Marco

Kyriakos Dimitriadis

Charilaos D Dimosthenopoulos

Tao Ding

Tayyab S Diwan

Priyanka Dixit

Carlota Dobaño Lazaro

Rachel Dockry

Lori Dodd

Matthew Dodd

Wolfram Doehner

Torsten Doenst

Noriko Doki

Geert Dom

YanHong Dong

Yanhui Dong

David A Dorr

Abigail Dove

Joseph Doyle

Lex Doyle

Luciano Drager

Catherine Draper

Alex Dregan

Jiangbo Du

Wenjie Duan

Rui Duarte

Mindy Duffourc

Graeme Duke

Rémy Duléry

Eric Durot

Julien Edeline

Fabio Efficace

Robert Eikelboom

Laya Ekhlaspour

Ahmed Elazab

Ibrahim El-Battrawy

Anthony Elias

Matilde Elices

Eric Emerson

Gideon Emukule

Yutaka Endo

Blake Erhardt-Ohren

Annette Erlangsen

María José Escámez

Gabriele Escherich

Tarık Esen

Ahmet Emre Eşkazan

Ciro Esposito

Mark Ettenberger

Ruth Etzioni

Paolo Eusebi

Laura Evangelista

Rachael Andrea Evans

James Fackler

Gian Paolo Fadini

Mark Faghy

Kristina Fahnehjelm

Adegoke Falade

Flaminia Fanelli

Eric Faragher

Michelle Farrar

Forough Farrokhyar

Martin Fassnacht

Seena Fazel

N Fazio

Deborah Fein

David Fei-Zhang

Reza Fekrazad

John Feldmeier

Yadong Feng

Giovanna Ferraioli

Marc Ferrante

Clarissa Ferrari

Montse Ferrer

Simone Ferrero

Rosario Ferrigno

Stefan Feuerriegel

Aaron J Fields

Guido Filler

Lucy Finnigan

Florian Fischer

Jane Fisher

Jennifer Flemming

David Flood

Damian G Fogarty

Pierre Foidart

László Földvári-Nagy

Lisa Force

Thomas Forst

Theodoros Foukakis

Stephanie Foulon

Mary Glenn Fowler

Chris Fox

Francesco Franceschi

Bruno Francois

Kate Frazer

Becky Freeman

Anna Freni Sterrantino

Pascal Frey

Ole Frobert

Le Fu

Hans Friedrich Fuchs

Marat Fudim

Jennifer Furin

Moritz Fürstenau

Maria Fusaro

Vittorio Fusco

Tonje Fyhn

Pierre-Henry Gabrielle

Gianluca Gaidano

Ognjen Gajic

Damien Galanaud

James Galloway

Habib Gamra

Venkatasubramanian Ganesan

Aravind Ganesh

Mohd Ganie

Ron Gansevoort

Andreas Rudolf Gantenbein

Chao Gao

Wei Gao

Miguel Garcia-Argibay

Jaime Garcia-Iglesias

Manuel Gardea-Resendez

Pankaj Garg

Ravindra Kumar Garg

Andrea Garolla

Emanuele Garreffa

Samvel B Gasparyan

Christopher Gast

Moshe Gatt

Kimberlee Gauvreau

Francesca Gay

Grace Gbotosho

Stefan Gebhardt

Jeroen Geldof

Jonathan Gelfond

James I Geller

Paul Gellert

Fulei Geng

Sommer E Gentry

Maureen R George

Puiu Lucian Georgescu

Raluca Georgescu

Bădicu Georgian

Marco Geraci

Patrick Gerardin

Georgios Germanidis

Lotte Gerritsen

Florian A Geßler

Wissam Ghusn

Valentina Giardini

Johannes Giesinger

Antonietta Gigante

Clare Gilbert

Antonio Gimeno-Miguel

Salvatore A Giordano

Adam W Glass

Nancy Glass

Louise Glenthoej

Bengt Glimelius

Katarina Glise Sandblad

Kathrin Gödde

Olivier Godefroy

Brian Godman

Matthias Goebeler

Amit Goel

Ralf Gold

Ronald Goldenberg

Jason Goldman

Sidra Goldman-Mellor

Jonathan Goldney

Delia Goletti

Jonathan Golub

Borja Gómez

David Gomez-Almaguer

Isidro Gonzales

Luis González-Bayón

Tomas Jose Gonzalez-Lopez

Alba González-Roz

Lakshmi Gopalakrishnan

Catherine Gordon

Johnathan Goree

Dzintars Gotham

Jiangtao Gou

Robert A Goulart

Antonios Gounaris Gounaris

Annabelle Gourlay

Vasantha Gowda

Alessandro Granito

Elin Gray

Raffaella Greco

Katie Greenzang

John Gregson

Lionel Gresh

Gulraj Grewal

Martin Grobusch

Kirsten Grønbæk

Petra Gronholm

Paolo Grossi

Sandeep Grover

Wan-Jie Gu

Wenyi Gu

Benoit Guery

Mireille Guillot

Andre Guimaraes

Nathalia Guimarães

Ruth Guinsburg

Nicholas Gulati

Roman Gulati

Martin Gulliford

Sinan Guloksuz

Jesper Gunst

Rui Guo

Wei Guo

Zhen-Ni Guo

Abha Gupta

Tejpal Gupta

Deborah Gustafson

J Antonio Gutierrez

Ruvistay Gutierrez-Arias

Bishal Gyawali

Bishal Gyawali

Allan Hackshaw

Danella Hafeman

Trish Hafford-Letchfield

Melanie Hagen

Wayne Hall

Rami Hallac

Yohhei Hamada

Mark Hamilton

William Hamilton

Jing Han

Qing Han

Xiangmin Han

Zhao Han

Gerard Hanna

Dalil Hannani

Michael Harhay

Arja Harila

Abdolreza Haririan

Julien Haroche

Diane Harper

Julie Harris

Lauren Harris

Kiyoshi Hasegawa

Kiyoshi Hasegawa

Cesare Hassan

Esraa Hassan

Md Mehedi Hassan

Michael Hawkes

Maria Hawkins

Hidetoshi Hayashi

Ping He

WenHua He

Yulei He

Audrey Hebert

Christel Hedman

Péter Hegyi

Thomas Hegyi

Lutz E Heide

Annie Heiderscheit

Carole Helissey

Thomas P Hellyer

Harri Hemila

Andrés Henao-Martínez

Michael Henderson

William Henley

Manuel Hernandez

David Hess

Jenny Hewsion

Martha Hickey

Jessica Hicks

Malene Grubbe Hildebrandt

Catherine Jane Hill

Fred R Hirsch

Gerard Hoek

Martin Hoenigl

Tomer Hoffman

Ulf Hogberg

Sarah Holmboe

Lisbet Hölmich

Caroline Homer

Andrew Hong

Myeong-Ki Hong

Lawrence C Hookey

Gary Hooper

Ashley Hopkins

Nir Horesh

Susan Horton

Aniqa Tasnim Hossain

Katsuyuki Hotta

Yu Hou

Hamidreza Houri

Jason Howard

Patricia Howlin

Chengyang Hu

Dongsheng Hu

Zhineng Hu

Dingzhi Huang

Yafang Huang

Yangmu Huang

M Mamun Huda

Tanvir Huda

Johannes Huebner

Karen Hughes

Siobhan Hugh-Jones

Robert Hüneburg

Ivan F N Hung

M Omair Husain

Waqar Husain

In-Chang Hwang

Zaza Iakobishvili

Luigi Iannone

Hirotoshi Iihara

Takaaki Ikeda

Tatsunori Ikemoto

Lawrence Impey

Manabu Inoue

Norimitsu Inoue

Elin Irestorm

Bilal Irfan

Sarina Isenberg

Hironori Ishigami

Kazuhiro Ishikawa

Arda Isik

Md Saiful Islam

Michael Ison

Yoshitaka Iwanaga

Sarah Jackson

Michael A Jacobs

Shabbar Jaffar

Mathieu Jagt

Ammar Abdulrahman Jairoun

Peter Jaksch

Matthew Jalink

Brian Jamieson

Daisy Janssen

Kajsa Järvholm

Shahrokh Javaheri

Reena V Jayani-Kosarzycki

David J Jenkins

Bec Jenkinson

Seogsong Jeong

Anders Jeppsson

Katarzyna Jerzak

Marc Gerhard Jeschke

Tine Jess

Nicholas Jewell

Rachel Jewkes

Bin Ji

Fanpu Ji

Muhuo Ji

Dong Jiang

Hongbo Jiang

Zhengyu Jiang

Jing Jin

Yuefei Jin

Zhi-Cheng Jing

Youngji Jo

Erika Jones

Ingibjörg H Jónsdóttir

Susan Jordan

K S Joseph

Anthony Joshua

Nathan Judd

Ivana Jukić

Steven Julious

Mikk Jürisson

Eliangiringa Kaale

Japhet Kabalu Tshiongo

Ashraf Kagee

Jan Willem Kallewaard

Yoichiro Kamatani

Kaloyan Kamenov

Richard Kanaan

Hyun Kang

Ellen Kapiteijn

Derin Karacabeyli

Paschalis Karakasis

Nuray A Karanci

Dulanjalee Kariyawasam

James Karl

R Jeffrey Karnes

Tilakavati Karupaiah

Diego Kaski

Gertjan Kaspers

Nicholas Kassebaum

Efstathios Kastritis

Preethy Kathiresan

Ken Kato

Aristeidis Katsanos

Michail Katsoulis

Morgan Katz

Kimito Kawahata

Mohammad Enamul Hoque Kayesh

Lu Ke

Josbert J Keller

Michael Patrick Kelly

Mark Kelson

Zoe Kelson

Elizabeth A Kelvin

Rick Kennedy

Rachel M Kenney

Louise A Keogh

Winfried Kern

W Kernohan

Stephen Kerr

Zafer Keser

Kenneth A Kesler

Ali Khashan

Minesh Khashu

Adree Khondker

Alexander Khoruts

Kamlesh Khunti

Arif Khwaja

Shoji Kido

Beom Kyung Kim

Hyungjin Kim

Hyung-Kwan Kim

Jaehong Kim

Tae Min Kim

William Taylor Kimberly

Shun-ichi Kimura

Ichiro Kinoshita

Paulus Kirchhof

Amar Kishan

Taro Kishi

Kosuke Kita

Ryan S Kitagawa

Ulla Klaiber

Marlena Klaic

Nicolas Kluger

Sven Knecht

Martin Kneyber

Johannes Knitza

Wen-Chien Ko

Silvia Kochen

Eiichi Kodama

Loick Pradel Kojom Foko

Kaija-Leena Kolho

Evangelos Kontopantelis

Sarah L Kopelovich

Marie Korhonen

Siran Koroukian

Ilma R Korponay-Szabó

Karel Kostev

Keita Kouzu

John Lawrence Kipling Kramer

Katharina Kranzer

Henry Kranzler

Clemens Kratochwil

Reinhold Kreutz

Eva Krockow

Karen Kroeker

Michael Krogsgaard

Nick Kruijt

Ilari Kuitunen

Anand Kulkarni

S Kunutsor

Yong-Fang Kuo

Hiroshi Kurita

Yukinori Kurokawa

Robert Kushner

Kwadwo Asamoah Kusi

Satoshi Kusuda

Mariusz Andrzej Kusztal

Tezer Kutluk

Satoshi Kuwabara

Cecilie Kyrø

Idoia Labayen

Karim Ladha

Chih-Cheng Lai

Julia Lai-Kwon

Sham Lal

Tania Lam

Wai Kei Jacky Lam

Christopher Lamb

Ning Lang

Jost Langhorst

Alice Laniepce

Francesco Lapi

Torben Larsen

Charlotta Larsson

Eric Lau

Kelsey Lau-Min

Jean Lawrence

Yael Lebenthal

Alexander Lee

Hong Lee

Jangming Lee

Jimmy Lee

Keng Siang Lee

Meihsuan Lee

Minji Kang Lee

Peter Lee

Sue Lee

Woojoo Lee

Sander Lefere

Rolf Lefering

Yang Lei

Yiting Lei

James Leigh

Jessica Leight

David Leon

Carl Leonhardt

William Leslie

Angela Leung

Kari Leung

Kathy Sze Man Leung

Thomas Leung

Jerrold Levy

Christelle Lévy

Daphne Lew

Roger J Lewis

Ben Li

Hong Li

Nan Li

Ni Li

Qingli Li

Sheyu Li

Weili Li

Xianjun Li

Xuehua Li

Yangqiu Li

Yi-Gang Li

Zhigang Li

Zhihui Li

Ioannis Liampas

Chih-Sung Liang

Fei Liang

Hengrui Liang

Wenhua Liang

Xiao Liang

Yannis Liang

Zhaoxia Liang

Zhongxing Liao

César R Libanati

Stafford Lightman

Soo Lim

Chin Lin

Ro-Ting Lin

Yansong Lin

Jacopo Lisoni

Anthony Pak-Yin Liu

Caigang Liu

Chenan Liu

Jiangang Liu

Jin Liu

Junting Liu

Lan Liu

Na Liu

Peter Y Liu

Qu Liu

Taotao Liu

Tong Liu

Xiaojuan Liu

Yueping Liu

Zhangsuo Liu

Zhenyu Liu

Zhen-zhen Liu

Zheran Liu

Alemneh Liyew

Frank Po Wen Lo

Carl Lombard

Fortunato Lombardo

Frédéric London

Michelle Look

Rubén López-Bueno

Jose Lopez-sendon

Florian Lordick

Yair Lotan

Paul Loubet

Alexander Louie

Bernd Löwe

Santiago Lozano-Calderon

Chan Lu

Liming Lu

Ming Lu

Qiang Lu

Mwansa Ketty Lubeya

Tim Luckett

Brandon Peter Lucke-Wold

Stefano Luminari

Fei Luo

Shuoming Luo

Xiangde Luo

Misha Luyer

Chaolan Lv

Han Lv

Yuebin Lv

Jun Lyu

Jun Ma

Xuelei Ma

Benjamin Maasoumy

Andrew Mackinnon

Graeme MacLennan

Alasdair MacLullich

Peter MacPherson

Finlay Alistair Macrae

Stefano Maria Magrini

Ashika D Maharaj

Maria Ida Maiorino

Lung-Yi Mak

Gin Malhi

Hassan Z Malik

David Malka

François Maltais

Abdullah Al Mamun

Katia Mancuso

Sema Mandal

Abhishek Avinash Mangaonkar

Lucia Mangone

Mattia Manica

Karim Manji

Brett Manley

J John Mann

Mohammad Ali Mansournia

Ning Mao

Xiaowei Mao

Natalie Marchant

David Joel Margolis

Georgios Antonios Margonis

Christophe Marguet

Thomas Maribo

Marco Marigliano

Marianne Martinello

Esteban Martinez

Nuria Martínez Cibrian

Flora Martinez Figueira Moreira

Pablo Martinez-Camblor

María Martínez-rebollar

Giovanni Martinotti

Daniel Fernandes Martins

Maria Beatriz Martins Linhares

Maura Massimino

Masayoshi Masuko

Arthur Matas

John Mathers

Yan Mathias Alves

Alexander G Mathioudakis

Prashant Mathur

Jordi A Matias-Guiu

Alexios Matikas

Isao Matsui

Elisa Mattavelli

Francesco Maura

Michael S May

Ismail Mayet

Finlay McAlister

Jessica McCann

Rachel D McCarty

Katrina McClannahan

Valerie McCormack

Natalie McCormick

Keith McCrae

Morgan McCreary

Gavin McDonnell

Kenneth McLean

Gearoid McMahon

Donald McMillan

Dulani Meedeniya

Stephanie Mehl

Rachana Mehta

Heng Mei

Zubing Mei

Samantha Meints

Mike Mendall

E F Mendonça

Claudia Mendoza-Pinto

Zhiqiang Meng

Fiona Mensah

Dick Menzies

Nicolas Menzies

Federico Mercolini

Maria Cristina Meriggiola

Tesfaye Mersha

Dominik Mertz

Alessio Mesini

Roberta Messina

Ludovico Messineo

Jonathan Metts

Alexander Meves

Patrick Meybohm

Gerd Meyer zu Horste

Navin Michael

Rod Middleton

Luca Miele

Filippo Migliorini

Stefan Mihaicuta

Tomi Mikkola

Freeman Miller

Nancy Hadley Miller

Edward Mills

Geltrude Mingrone

Vincent Minville

Charles Mitchell

Katy Mitchell

Sarah Jane Mitchell

Hiroshi Miyamoto

Ryo Miyata

John Modlin

Frederick Gerard Moeller

Ghulam Mohyuddin

Riccardo Moia

Alessio Molfino

Ghyslain Mombo-Ngoma

Gemma Moncunill

Guillaume Monneret

Carlos Monteiro

Chris Monten

Maria Cecilia Montenegro

Francesco Montorsi

Catrin Moore

Alessandro Morandi

Raul Moreno

Victor Moreno

Harriet Morf

Catherine Morgan

Abraham Morgentaler

Hiroyoshi Mori

Norman R Morris

Rachel A Moses

Ari L Moskowitz

Barbara Mostacci

Sizulu Moyo

Mwenya Mubanga

Maria Lorenza Muiesan

Fabian Müller

Erin E Mulvihill

Hassan Mumtaz

Jennifer Murphy

Shilpa Murthy

Dildar Haji Musa

Cristina Mussini

Godfrey Nyangadzayi Musuka

Christine Wayua Musyimi

Jeffrey Leon Myers

Paul Myles

Ernest Nadal

Peter Nagele

Tateaki Naito

Yousuke Nakai

Akinobu Nakamura

Eve Namisango

George E Naoum

Abdulqadir Nashwan

Peiman Nazerian

Paul Nederkoorn

Eva Negri

Ashley M Nelson

Karin M Nelson

Lindsay Nelson

Nicolas Nesseler

James Neuberger

Alfred Neugut

Patrick Neven

Qin Xiang Ng

Preston Ngo

Huong Nguyen

Tacilta Nhampossa

Jianjiao Ni

Anthony Nichols

Mark Nicol

Runcong Nie

Sheng Nie

Niklas Nielsen

Lourdes Nieto

Hiromichi Niikawa

Riitta A Niinimäki

Inger S Nijhof

Anton Nikouline

Linn Nilsen

Yuye Ning

Takeshi Nishimura

Dorit Nitzan

Dorit Nitzan

Basile Njei

Kentaro Noda

Natalie Nokoff

Marianne Nordsmark

John Norrie

Jesper Nors

Seyed Mehdi Nouraie

Ashraf Noureldin

Iona Novak

Silvia Nozza

Rui Nunes

Isaac Nuñez

John L Z Nyirenda

Alexander Nystrom

Reginald Obiako

Myles O'Brien

Stewart O'Callaghan

Chan-Young Ock

Kazutaka Oda

Katherine Odem-Davis

Camila Odio

Owen O'Donnell

Sabine Oertelt-Prigione

Yoshihiro Ogawa

Robin Ohls

Christian Ohmann

Kazuo Ohtsuka

Christoph Oing

Masato Okada

Alicia Okines

Cecilia Okusi

Ayako Okuyama

Laura J Olivieri

Henrik Olsen

Ali Omrani

David O'Neal

Francesca Felicia Operto

Randi Opheim

Katrina Ortblad

Edgar Ortiz-Brizuela

Augustus Osborne

Piet Ost

Marie Österberg

Marlies Ostermann

Dylan O'Sullivan

Nancy Otieno

Willem Otte

Rodolfo J Oviedo

Carolyn J Owen

Yukinori Ozaki

Gozde Ozakinci

Robert Paine III

Bruno Paiva

Enny Paixao

Janini Paiz

Eva Pajkrt

Nirianne Marie Palacpac

Philip Pallmann

Jaana Paltamaa

Huaqin Pan

Sanjay Pandanaboyana

Vitor Hugo Panhóca

Georgios Panos

Konstantinos Papamichail

Antoine Pariente

Jean-Jacques Parienti

Sunil Parikh

Bomi Park

Donald Parkin

Avinash Parnandi

H Roeline Willemijn Pasman

Patrizio Pasqualetti

Sandro Pasquali

Giuseppe Passarino

Eshan Patel

Roshan Paudel

Mical Paul

Martin Paulus

Lillian Pazvakawambwa

Fedro Peccatori

Salvador Peiró

Päivi T Peltomäki

Sarah Pendlebury

Junjie Peng

Zhiyong Peng

Pietro Pepe

Carlo Federico Perno

Anne Perozziello

Meredith Perry

Gianluca Perseghin

Maiju Pesonen

Jonathan Peter

Olumuyiwa James Peter

Trevor Peter

Petra Petranovic-Ovcaricek

Max Petrov

Per Pfeiffer

Paul D P Pharoah

Bob S Phillips

Wiraphol Phimarn

Maria Carmela Piccirillo

Suzanne Pickering

Samuel Pierre

Albrecht Piiper

Mershen Pillay

Jennifer Pillow

Terhi Piltonen

Patricia Piña-Sánchez

Paul Pinsky

Punnee Pitisuttithum

Bertram Pitt

Milan Piya

Jennifer Plichta

Sven Poli

Constance Dimity Pond

Gregory Pond

Bernard Pope

Allison Portnoy

Regina Poß-Doering

Neelam Potdar

Philip A Powell

Melanie Powis

Anton Pozniak

Asta Ratna Prajapati

Mandi Pratt-Chapman

Meir Preis

Aleksander Prejbisz

Kiesha Prem

Robin Prescott

Laura Prichett

Jan Prins

Jerilynn Prior

Are Hugo Pripp

Kathy Pritchard-Jones

Susan Prockop

M H Proust

Adam Przybyłkowski

Fabio Puglisi

Michael Pulia

Ibrahim Qaddoumi

Qibin Qi

Xiaowei Qi

Guanghui Qian

Jianfeng Qiu

Jiliang Qiu

Yun Qiu

Niels Qvist

Karen Rabin

Karen Rabin

Lukas Radbruch

Venkatraman Radhakrishnan

Preeti Raghavan

Giovanni Raimondo

Anupama Rajanbabu

Kaushik Ramaiya

Mika Ramet

Vundli Ramokolo

Aaron Rapoport

Sotiris Raptis

Emanuel Raschi

Corey Ratcliffe

Navid Razmjooy

Chakradhar M Reddy

Julie Redfern

Jill Reedy

Annette Regan

Orna Reges

Dag Henrik Reikvam

Jiansong Ren

Shaolong Ren

Domenico Rendina

Rungsun Rerknimitr

Anna Reyners

Andrew Reynolds

Simone Ribero

Rosemary Ricciardelli

Matteo Riccò

Debra Richardson

Thomas Richie

Hannah Rickman

Kathryn Ridout

Stefan Rieken

Lorenza Rimassa

Andreas Rimner

Anamara Ritt-Olson

Alessandro Rizzo

Jared Rex Robbins

Lawrence R Robinson

Marco Rocchi

Anne Rochtus

David Rodeberg

Francisca Rodriguez

Leonardo Roever

Mary-Louise Rogers

Eliane Rohner

Philippine (Pip) Roijer

Alexandra Roldan

Ingmar Florin Rompen

Rafael Rosell

Joshua Rosenblat

Gholamreza Roshandel

Allen Ross

Jennifer Rossen

Peter Rossing

Micol Rothman

Giandomenico Roviello

Peter Rowe

Debananda Roy

Patrick Rozenberg

Yibing Ruan

Christian Ruck

M David Rudd

Piero Ruggenenti

Sebastian Rühling

Yun-Feng Rui

Luis Ruilope

Mihir Prafulbhai Rupani

Piero Ruscitti

Stefano Rusconi

Marc Russo

Piotr L Rutkowski

Frans Rutten

Ricardo Ruttimann

Lisa Rydén

Suresh Anand Sadananthan

Ayoub Saeidi

Marc Saez

Ranjit Sah

Bita Sahaf

Daljit Singh Sahota

Brodie M Sakakibara

Tomohiro Sakamoto

Elisa Sala

Zikria Saleem

Noemi Salmeri

Serena Salome

Ray Samuriwo

Nuria Sanchez Clemente

Andres Sánchez-Pernaute

L Nelson Sanchez-Pinto

Diana C Sanchez-Ramirez

Robert David Sanders

Lisa Sandmann

Ingvild Sandoy

Fuat Hakan Saner

Takeshi Sano

Babak Sarani

Celestino Sardu

Jerome Sarris

Kazunari Sasaki

Sharon H Saydah

Alyssa Sbarra

Lorenzo Scappaticcio

Benedikt Schaefer

Lidia Schapira

Thomas Scharschmidt

Douglas Schaubel

Edward Schenck

Georg Schett

Chris G Schilling

Michèle M Schlehofer

Sabrina Schlesinger

Wolf Schmidt

Martin A Schneider

Melissa J Schoelwer

Nancy Li Schoenborn

Reineke Schoot

Christina Schroeter

Johannes Kurt Schultz

Jonathan Schulz

Ulrike Schulze

Mark Schuuring

Lee S Schwartzberg

Nora Schwotzer

Francesco Sclafani

Natasha Seaton

Barbara Joyce Seaworth

Ilana Seff

Akihiro Seita

Thomas Sejersen

Shahjada Selim

Thiviya Selvanathan

Carlo Senore

Sadaf Sepanlou

Denis Sereno

Shekhar Seshadri

Ahmet Sezer

Muhammad Shaban

Muhammed Shabil

Gunjan Shah

Muhammad Aaqib Shamim

Michel Shamy

Peng-Fei Shan

Oliver Shannon

Shih-Chieh Shao

Iraj Sharifi

Arya Sharma

Zainab Shateri

James Shaw

Jonathan Shaw

Kathryn Shaw-Saliba

Yunlang She

M Kyla Shea

Joseph Shearer

Yahya Shehabi

Jun Shen

Yueping Shen

Susan Deborah Shenkin

Vicky Sheppeard

Alexander Sherry

Han-Ping Shi

Ju-Fang Shi

Qiang Shi

Norihisa Shigemura

Shouchuan Shih

Masayuki Shima

Eiji Shimizu

Masayoshi Shinjoh

Kohei Shitara

Shmuel Shoham

Dhananjay Shukla

Lu Si

Weixin Si

Spyridon Siafis

Michael J Sichlau

Ilias Siempos

Maria Silveira

David Simmons

Kate Simms

Michael Simon

Colin Simpson

John Simpson

Mervyn Singer

Bhajan Singh

Prabhishek Singh

Siddharth Singh

Pranay Sinha

Andrew Sinnamon

Vassiliki Sinopoulou

Regina Sit

Parco Siu

Pradeesh Sivapalan

David Sjöström

Becky Skidmore

Nicole Skoetz

Lone Skov

Roman Škulec

Marry Smit

Jeffery Smith

Jesse Joshua Smith

Stephanie Smith

Thomas Smith

Joseph Smolich

Luís Eduardo Silva Soares

Johan Söderholm

Shahrul Aiman Soelar

Mette Søgaard

Virginia Solitano

Luba Sominsky

Vernon Sondak

Jingchun Song

Mingyang Song

Yi Song

Milan Sonneveld

Guru Sonpavde

Antoni Soriano-Arandes

Alvaro Sousa

Kevin W Southern

Joshua P Spaete

Dorothee Speiser

Andrew Spillane

Alicia Spittle

Daniel Spratt

Chandrashekhar Sreeramareddy

Anand Srinivasan

Fabio Stagno

Dan Stark

Luca Steardo Jr

Philip J Steer

Pasquale Stefanizzi

Daniel Steffens

Bernhard Steinhoff

Jacob Steinle

Timothy Sterling

Barney Stern

Michael Sticherling

Charles Stiller

Christian Stoppe

Stefan Störk

John Stover

Rupert Wolfgang Strauss

Sam Straw

Franc Strle

Jonathan Strosberg

Michael Strupp

Paula Suárez-Pinilla

David Sugerman

Xinbing Sui

Richard Sullivan

Lidan Sun

Karin Sundström

Sujatha Sunil

Aditya Surapaneni

Bernard Surial

Robert Sutcliffe

Matthew Sydes

Christos Symeonides

Tamas Szakmany

Stanley Szefler

John Szumowski

Marina Tadolini

Mahdi Taghadosi

Heidi T Taipale

Kashihara Tairo

Nozomi Takahashi

Satoshi Takanashi

Tomofumi Takaya

Hiroyoshi Takeuchi

Xiao Tan

Atsushi Tanaka

Kentaro Tanaka

Jie Tang

Julian Tang

Li Tang

Lin Tang

Ling-Long Tang

Pieter Tanis

Bachir Taouli

Hirotaka Tashiro

Chau Thien Tay

Nicholas F Taylor

Rod S Taylor

Marleen Temmerman

Jaap ten Oever

Shu Mei Teo

Lauren Teras

Pauline D Terebuh

Vito Terlizzi

Massimo Terzolo

Benoît Tessoulin

Brian Thoma

John Graham Thomas

Philip A Thompson

Mette K Thomsen

Claire Thorne

C Thwaites

Beth Tippett-Barr

Sofonyas Abebaw Tiruneh

Matthias Titeux

Gabriela Toledo

Antonio Toniolo

Jorma Toppari

Julie S Torode

Josie Resende Torres Da Silva

Francesco Torri

Iakovos Toumazis

Marcos Roberto Tovani-Palone

Hidenori Toyoda

Adrian Traeger

Thach Tran

Thi Xuan Mai Tran

Viet-Thi Tran

Hop Tran Cao

Jonel Trebicka

Logan Trenaman

Eileen Trigoboff

Antoni Trilla

Stephanie Truant

Ivan Trus

Yuan-Ming Tsai

Georgios Tsivgoulis

Nobuo Tsuboi

Eiichi Tsuda

Kunihiro Tsukasaki

Shinya Tsuzuki

Mehmet Turgut

Richard Turner

Arnljot Tveit

Gilad Twig

William R Tyor

Shinichiro Uchiyama

Ugochinyere Vivian Ukah

Niels Uldbjerg

Emre Umucu PhD

Luca Urso

Krishna Mohan Vadrevu

Alex Valadka

Alexander J C van Akkooi

Rombout van Amstel

Sari van Anders

Marleen Van Baak

Eric van Belle

Jan Willem Van Dalen

Andry Van de Louw

Roderick C N Van Den Bergh

M Beatrijs van der Hout-van der Jagt

Hendrik van der Worp

Joris van Dongen

H Rogier van Doorn

Nielka P van Erp

F J van Kemenade

Elisabeth van Leeuwen

Max van Noesel

Gabrielle van Ramshorst

Ashlee J Vance

Paul Van-Schaik

David Noel Vaughan

Lennert Veerman

Sithembiso Velaphi

Vincenzo Venerito

Francois Venter

Geert Verleden

Ashish Verma

Fabienne Vervaat

Marie Vidailhet

Radana Vilimkova Vilimkova Kahankova

Guillermo Villacampa

Andrea Visentin

Leo Visser

Vijay Viswanathan

Antonio Vita

Michele Vitacca

Giulia Vivaldi

Simon Völkl

Lena von Bahr

Bernd Vorderwülbecke

Vladan Vucinic

Sudheer Vuyyuru

Anja Wagner

Gerd Wagner

Amy E Wahlquist

Robert Walker

Jack Wallace

Robert Bruce Wallace

Eugene Haydn Walters

Seth Wander

Chengdi Wang

Chi Chiu Wang

Ching-Chia Wang

Fu-Sheng Wang

He Wang

Hexiang Wang

Hsiang-Chen Wang

Huanjun Wang

Huanmin Wang

Huilin Wang

Jaeyun Jane Wang

Jiwei Wang

Junxiao Wang

Kai Wang

Kun Wang

Mengzhao Wang

Peng-Hui Wang

Qian Wang

Qiming Wang

Qinchuan Wang

Tiange Wang

Wei Wang

Xin Wang

Xinning Wang

Xinying Wang

Yi Wang

Yu Wang

Yu Wang

Yuang Hung Wang

Zhu Wang

Heather Ward

Thomas Ward

Jennifer Warnick

Fiona E Watt

Gerald Watts

Lindell Weaver

Marcia Weaver

Heather E Webber

Joseph Wee

Lachelle Weeks

Chen Wei

Alain Weill

Adriana Weinberg

Martin Welcker

André Werneck

Bernhard Wernly

Steven Wexner

Mark Whiteley

Gary Whitlock

Luuk Wieske

Alexander Wilhelm

Malgorzata Wilinska

Shelley Ann Wilkinson

Anita Williams

Linda Williams

Tom Wingfield

Stephen Wisniewski

Nicole Wolf

Grace Wong

Ka-Chun Wong

Robert Wong

Theodoric Wong

Rachel C Wood

Gillian Worthy

Clinton Wright

Paul Wright

Chin-Chieh Wu

Depei Wu

Jiayuan Wu

Lin Wu

Qi-Jun Wu

Runhui Wu

Xiang Wu

Xiaodong Wu

Xifeng Wu

Karen H Wynter

Piotr Wysocki

Mian Xi

Yang Xia

Yankai Xia

Gaosi Xu

Huiping Xu

Long Xu

Min Xu

Sanzhong Xu

Shouping Xu

Wanghong Xu

Xiaolin Xu

Xiaopan Xu

Yu Xu

Yun Xu

Ziyan Xu

Keisuke Yaku

Takayuki Yamamoto

Hajime Yamazaki

Bo-Yi Yang

Guozi Yang

Hanxue Yang

Lawrenceh Hsin Yang

Li Yang

Lin Yang

Min Yang

Shigui Yang

Xiaorong Yang

Xue Yang

Zhirong Yang

Noam Yarom

Ding Ye

Junzhao Ye

Yung-Hsin Yeh

Nadir Yehya

Minyue Yin

Haochao Ying

Terry Yip

Kai-Hang Yiu

Tomoya Yokota

Naohiro Yonemoto

Paul J Yong

Wei Peng Yong

Hiroshi Yoshida

Yusuke Yoshikawa

Paul Young

Clare Yu

Honggang Yu

Honggang Yu

Huiting Yu

Ke-da Yu

Ming-Lung Yu

Yongfu Yu

Tarik K Yuce

Man Fung Yuen

Atil Yüksel

Donghwan Yun

Kai Zacharowski

David Zakim

Hongmei Zeng

Dongfeng Zhang

Fengmin Zhang

Fengqing Zhang

Guijun Zhang

Huabing Zhang

Jie Zhang

Jingsong Zhang

Li Zhang

Lin Zhang

Lina Zhang

Rui Zhang

Siwen Zhang

Wensheng Zhang

Xiaodong Zhang

Xiaoming Zhang

Yan Zhang

Yifeng Zhang

Yiqun Zhang

Yong Zhang

Zhen Zhang

Zhen Zhang

Zhongheng Zhang

Chongke Zhao

Dongchi Zhao

Fang-Hui Zhao

Fengmin Zhao

Jie V Zhao

Jiuda Zhao

Qingguo Zhao

Shi Zhao

Yanlin Zhao

Yuan-Yuan Zhao

Jie Zheng

Ming-Hua Zheng

Yijuan Jun Zheng

Zhiyuan Zhong

Chong Zhou

Guoren Zhou

Qing Zhou

Shiyu Zhou

Shouhao Zhou

Xin Zhou

Xu-jie Zhou

Yushy Zhou

Zeping Zhou

Bin Zhu

Hui Zhu

Zezhang Zhu

Zhengfei Zhu

Lauren Ziemba

Tommaso Zoerle

Edward Zoratti

Mohamed Ali Zoromba

Yutian Zou

Zhiyong Zou

Cheng Zu

